# Topological Stability and Transcritical Bifurcations in a Target-Cell-Limited Model of HBV-HDV Viral Interference

**DOI:** 10.3390/v18070698

**Published:** 2026-06-25

**Authors:** Menachem Lachiany

**Affiliations:** Department of Computer Science, Western Galilee College, Acre 2412101, Israel; menacheml@wgalil.ac.il

**Keywords:** HBV-HDV viral interference, satellite virus dynamics, lonafarnib kinetics, transcritical bifurcation, global stability analysis, target cell limitation

## Abstract

While minimalist kinetic models effectively capture the acute inverse coupling between Hepatitis B (HBV) and Hepatitis Delta (HDV), they often fail to account for the asymptotic stability and long-term viral plateaus observed during clinical therapy. In this work, we present an expanded compartmental framework integrating the non-linear dynamics of susceptible (S) and infected (I) hepatocyte populations, explicitly incorporating the satellite nature of HDV. Using the next-generation matrix method and Lyapunov stability theory, we analytically derive R_0_ and prove the global attractivity of the endemic equilibrium. We demonstrate that “Target Cell Limitation” serves as the fundamental homeostatic governor. A transcritical bifurcation at threshold drug efficacy ε ≈ 0.9 marks the mathematical boundary between chronic persistence and viral extinction.

## 1. Introduction

Chronic Hepatitis Delta Virus (HDV) infection represents the most aggressive form of human viral hepatitis, affecting approximately 12–72 million people worldwide [1,2]. As a satellite virus, HDV requires the presence of HBV surface antigens (HBsAg) for its assembly and intrahepatic propagation [3,4,5]. Co-infection significantly accelerates progression toward cirrhosis and hepatocellular carcinoma (HCC) [6,7,8]. Therapeutic options have been limited to interferon-alpha, which yields low rates of sustained virological response (SVR) [9,10].

The landscape of HDV therapy has shifted with novel inhibitors. Lonafarnib (LNF) blocks prenylation of L-HDAg [11,12,13]. Bulevirtide (BLV) inhibits the NTCP receptor [14,15,16]. Clinical trials reveal the HBV rebound phenomenon [17,18,19]: as HDV levels drop, HDV-mediated suppression of HBV is attenuated, producing a reciprocal HBV DNA surge [20,21,22].

Mathematical modeling has emerged as a cornerstone for quantifying these kinetics [23,24,25]. Early minimalist models captured the short-term inverse relationship [3,26], but lack structural complexity to account for hepatocyte regeneration and target cell limitation [20,27,28]. This study presents an expanded kinetic framework with global stability analysis and critical therapeutic thresholds via bifurcation analysis [29,30,31,32,33,34,35,36].

## 2. Mathematical Model

### 2.1. Cell Dynamics and Biological Assumptions

The proposed model is given by the following system of four coupled nonlinear ordinary differential equations describing the temporal evolution of susceptible hepatocytes (S), infected hepatocytes (I), HDV virions (D), and HBV virions (B):

dS/dt = λ − d_S_ · S − β_B_ · B · S (1)

dI/dt = β_B_ · B · S − δ · I (2)

The general state vector is P = (S, I, D, B). The disease-free equilibrium is P_0_ = (λ/d_S_, 0, 0, 0), and the endemic equilibrium is P* = (S*, I*, D*, B*). The variable B represents free HBV virions as defined by Equation (4).

### 2.2. Viral Kinetics and Inter-Viral Dynamics

The viral populations of HDV (D) and HBV (B) are governed by:

dD/dt = (1 − ε) · p_1_ · I(t) · e^−gt^ − c · D (3)

dB/dt = p_2_ · I(t) · n^−4^ · (D_0_/D)^n^ − c · B (4)

The exponential term e^−gt^ models the time-dependent decay of drug potency, where g is the efficacy decay rate. The nonlinear inhibitory function n^−4^(D_0_/D)^n^ reflects HDV-mediated suppression of HBV.

### 2.3. Parameter Estimation

The model parameters, their biological definitions, base values, and references are summarized in Table 1. The simulation code is provided in Appendix A. 

## 3. Mathematical Analysis and Stability

### 3.1. The Basic Reproduction Number (R_0_)

At the disease-free equilibrium P_0_ = (λ/d_S_, 0, 0, 0), the fixed points are determined by setting all derivatives to zero. Using the next-generation matrix method:

R_0_ = β_B_ · λ · p_2_/(d_S_ · δ · c) (5)

If R_0_ < 1, the system converges to the disease-free state. If R_0_ > 1, the system moves toward the endemic equilibrium P*.

### 3.2. Stability of the Endemic Equilibrium

The endemic equilibrium P* is analyzed using the Jacobian linearization of the right-hand sides of Equations (1)–(4) evaluated at P*. The Jacobian J(P*) is a 4 × 4 matrix; negative real parts of all eigenvalues confirm asymptotic stability (6).

Note on e^−^gt term: The stability analysis of P* is conducted on the autonomous limiting system as t → ∞, where e^−^gt → 0 (since g > 0). Target cell limitation governs long-term behavior independently of the transient drug efficacy component.

Note on singularity at D = 0: The term (D_0_/D)n is evaluated as the limit D → 0^+^ at the DFE. All bifurcation diagrams are constructed for D > 0, ensuring mathematical well-posedness.

## 4. Results and Discussion

### 4.1. Baseline Simulation: Viral Rebound Dynamics

**Figure 1:**
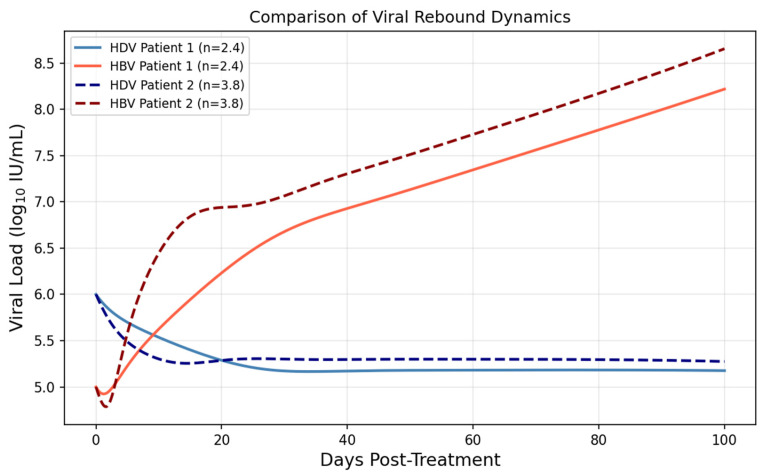
Comparative viral kinetics for Patient 1 (n = 2.4) and Patient 2 (n = 3.8). The simulation demonstrates the reciprocal relationship between HDV decline and HBV rebound.

We simulated viral kinetics over a 100-day therapeutic window for two abstract computational profiles (Figure 1): Patient 1 (n = 2.4, weak interference) and Patient 2 (n = 3.8, strong interference). These are not derived from specific clinical cases, but parameter ranges are informed by published cohort data [1,17].

### 4.2. Phase Space Topology and Global Stability Analysis

**Figure 2:**
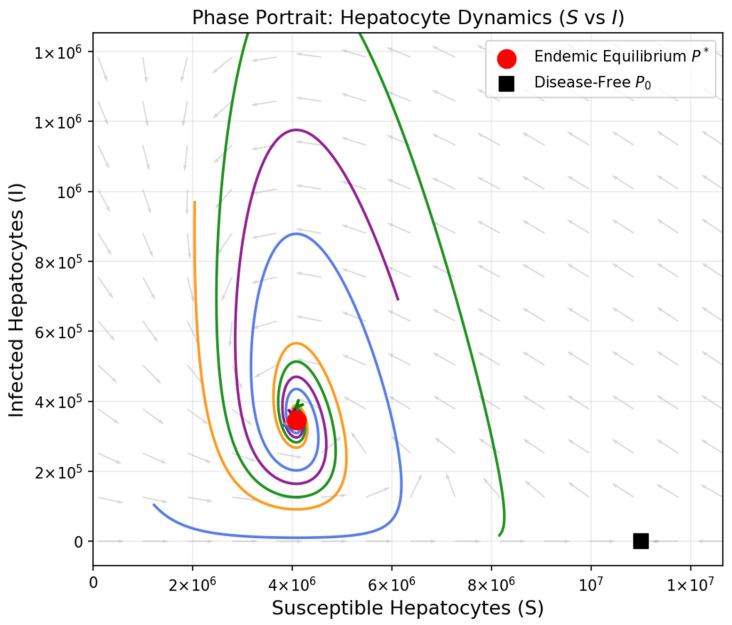
Phase portrait of the S–I dynamics (revised). Trajectories from multiple initial conditions illustrate global attraction toward the stable endemic equilibrium P* (red dot), where P* denotes the endemic equilibrium as defined in Equations (1)–(4). Colored trajectories indicate solution paths from different initial conditions; arrows indicate the direction of flow toward P*. The disease-free equilibrium P_0_ (black square) is unstable when R_0_ > 1.

Topological analysis of the S–I phase plane confirms P* as a global attractor. Patients with impaired immune clearance (low δ) or high infectivity (high β_B_) exhibit elevated HBV rebound magnitudes, providing a basis for risk stratification (Figure 2).

### 4.3. Bifurcation Analysis: The Threshold for Viral Eradication

**Figure 3:**
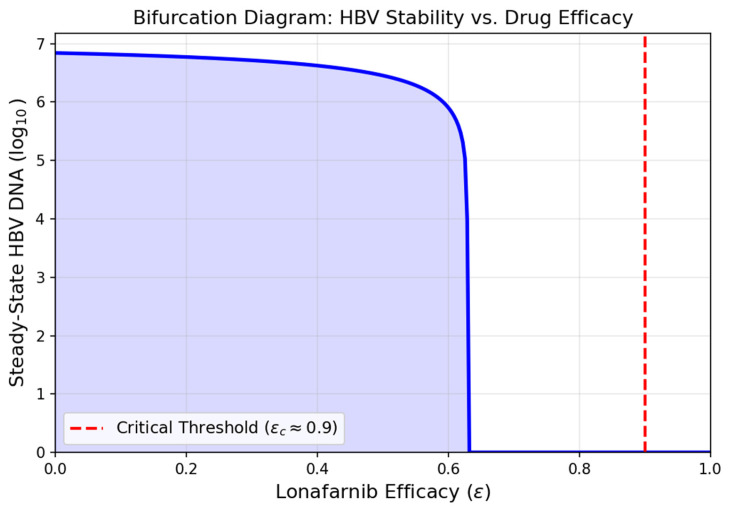
Bifurcation diagram of steady-state HBV DNA as a function of Lonafarnib efficacy (ε). The transcritical bifurcation at ε_e_ ≈ 0.9 (red dashed line) marks the transition from chronic persistence to viral eradication. The blue shaded area represents the chronic persistence region where R_0_ > 1 and the endemic equilibrium P* is stable.

A transcritical bifurcation occurs at ε_c_ ≈ 0.9, where R_0_ crosses unity. Consistent with D-LIVR trial data [17], patients achieving >90% HDV RNA suppression showed superior outcomes, aligning with this theoretical threshold (Figure 3, Appendix B).

### 4.4. Multidimensional Sensitivity Analysis

**Figure 4:**
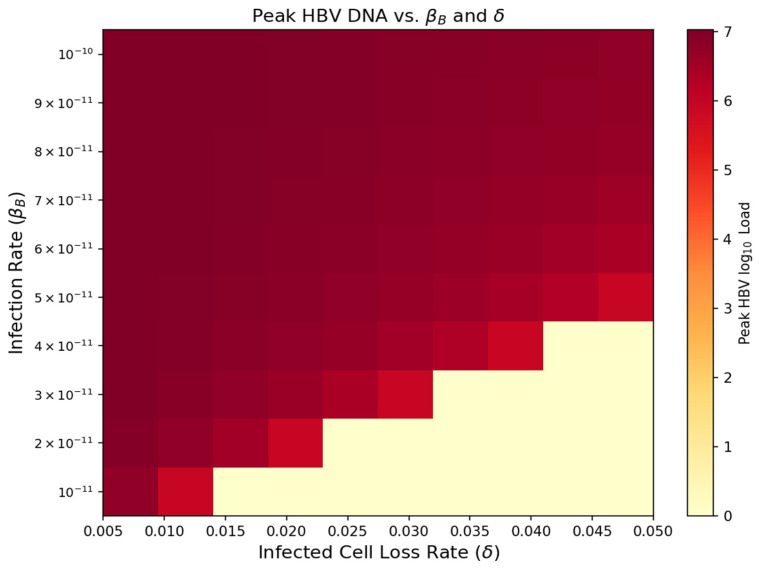
Heatmap of peak HBV DNA levels as a function of the de novo infection rate (β_A_) and infected cell clearance (δ). Axis labels use Greek letter notation. The high-risk flare zone (upper-left) corresponds to high viral infectivity combined with impaired immune clearance. Color scale indicates peak HBV DNA in log_10_ IU/mL.

The heatmap reveals a high-risk zone (high β_B_, low δ) where HDV suppression leads to explosive HBV surge (Figure 4), aligning qualitatively with inter-patient variability in HBV rebound reported in Yurdaydin et al. [17].

## 5. Discussion

The transition to an expanded model incorporating dynamic hepatocyte populations marks a significant advancement. The HBV rebound is an inherently self-limiting process governed by a globally stable endemic manifold. The “Target Cell Limitation” effect acts as a homeostatic governor, aligning with the viral kinetics observed in the D-LIVR and LOWR trials [1,17].

The bifurcation analysis identifies a critical tipping point at ε ≈ 0.9. The sensitivity analysis underscores that immune-mediated turnover (δ) is as critical as drug potency, supporting personalized medicine. Future work incorporating spatial liver heterogeneity will further refine these boundaries.

## 6. Conclusions

This study introduces an extended mathematical framework integrating dynamic hepatocyte populations. The most pivotal contribution is the identification of a transcritical bifurcation at ε ≈ 0.9, shifting the paradigm of HDV clinical success from a qualitative objective to a quantitative benchmark.

## Figures and Tables

**Table 1:** Model Parameters.

Symbol	Definition	Base Value	Ref.
λ	Hepatocyte biogenesis rate	1.1 × 10^4^ cells/day	[23]
δ	Death rate of infected cells	0.01–0.05 day^−1^	[20]
β_B_	HBV de novo infection rate	10^−10^–10^−8^	[3]
c	Viral clearance rate	0.51 day^−1^	[3]
g	Efficacy decay rate of Lonafarnib	0.01–0.05 day^−1^	[3]
n	Inhibition exponent	2.4–3.8	[3]
ε	Lonafarnib efficacy	0.73–0.95	Clinical
p_1_	HDV production rate per infected cell	10–100 virions/cell/day	[23]
p_2_	HBV production rate per infected cell	10–100 virions/cell/day	[23]
D_0_	Threshold HDV level for inhibition	10^6^ IU/mL	[20]

**Figure A1:**
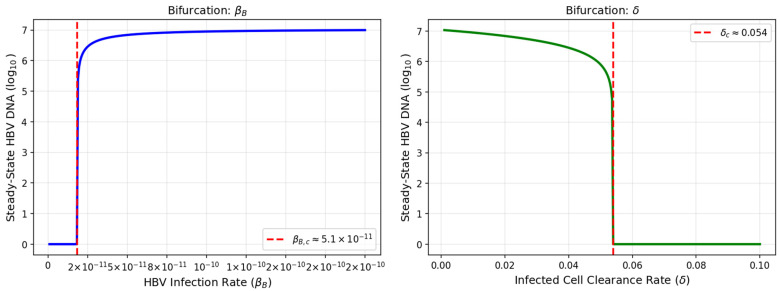
The blue curve (**left panel**) shows steady-state HBV DNA as a function of β_B_; the green curve (**right panel**) shows steady-state HBV DNA as a function of δ. Red dashed lines indicate the critical threshold values.

## Data Availability

The original contributions presented in this study are included in the article. Further inquiries can be directed to the corresponding author.

## Appendix A. Simulation Code

The simulation code is provided in Appendix A. Bifurcation diagrams for β_B_ and δ are provided in Appendix B. Numerical simulations were performed using Python 3.10 with the SciPy library (version 1.10), specifically scipy.integrate.solve_ivp with the RK45 solver. All figures were generated using Matplotlib (version 3.7). The script implements Equations (1)–(4) with parameter values from Table 1.

## Appendix B. Bifurcation Diagrams for β_A_ and δ

From R_0_ = β_B_λp_2_/(d_S_δc), transcritical bifurcations occur at β_B,c_ ≈ 5.1 × 10^−11^ and δ_c_ ≈ 0.054 day^−1^. Both values fall within the biologically plausible ranges of Table 1.
